# Case report: Orbital dirofilariasis

**DOI:** 10.4103/0971-3026.37050

**Published:** 2008-02

**Authors:** M Smitha, VR Rajendran, E Devarajan, PM Anitha

**Affiliations:** Departments of Radiodiagnosis and Microbiology, Medical College, Kozhikode, Kerala, India

**Keywords:** CT, dirofilariasis, orbital, USG

Dirofilariasis is a worldwide zoonotic filariasis with over 782 cases reported so far from different parts of the world.[1] Of these, about a third affects the orbit. However, the imaging features of orbital dirofilariasis are not well described in literature, with the diagnosis usually being made only after surgical excision for suspected orbital tumor. On reviewing the literature we found that no cases of orbital dirofilariasis diagnosed by USG have been reported so far. We would like to report the case of a 40-year-old Asian Indian woman with orbital dirofilariasis.

## Case Report

**Figure 1:**
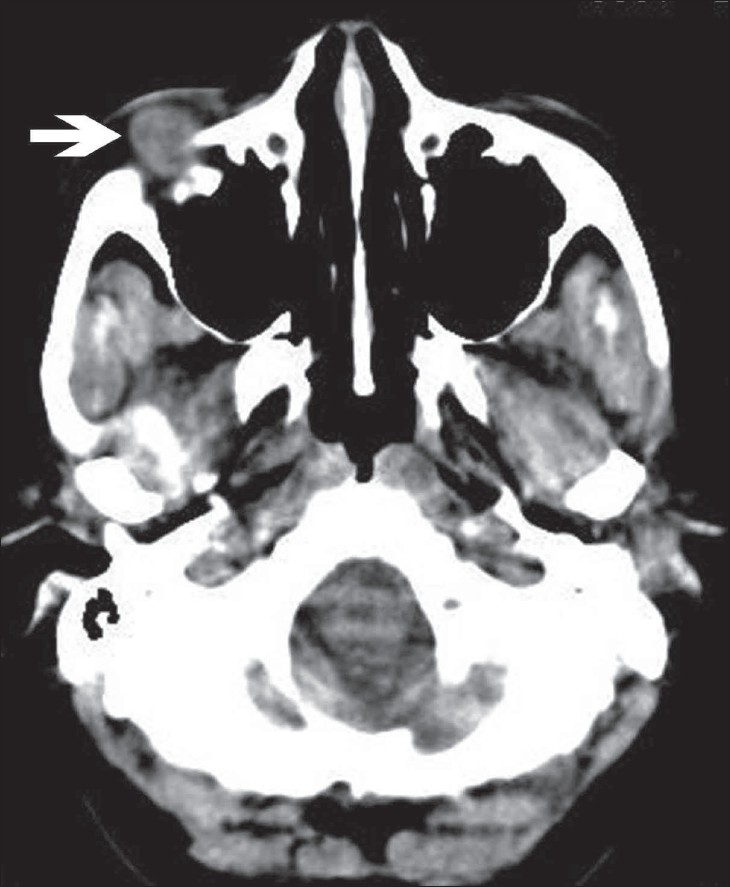
Plain axial CT image of the orbit showing a well-defined, heterogeneous lesion in the right orbit, extraconal in location (arrow)

**Figure 2:**
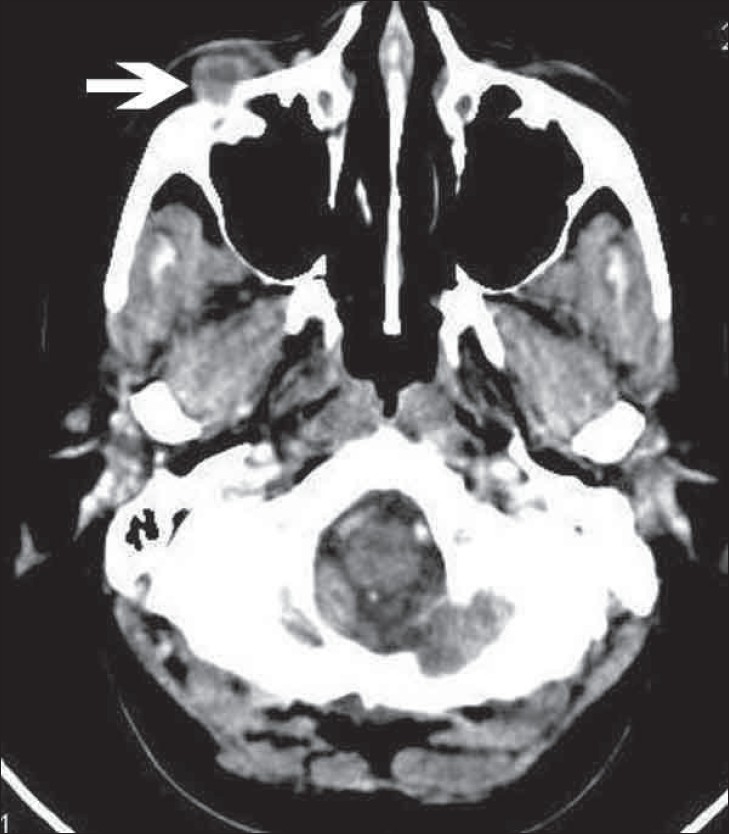
Post-contrast axial CT image showing heterogeneous enhancement of the lesion (arrow)

**Figure 3:**
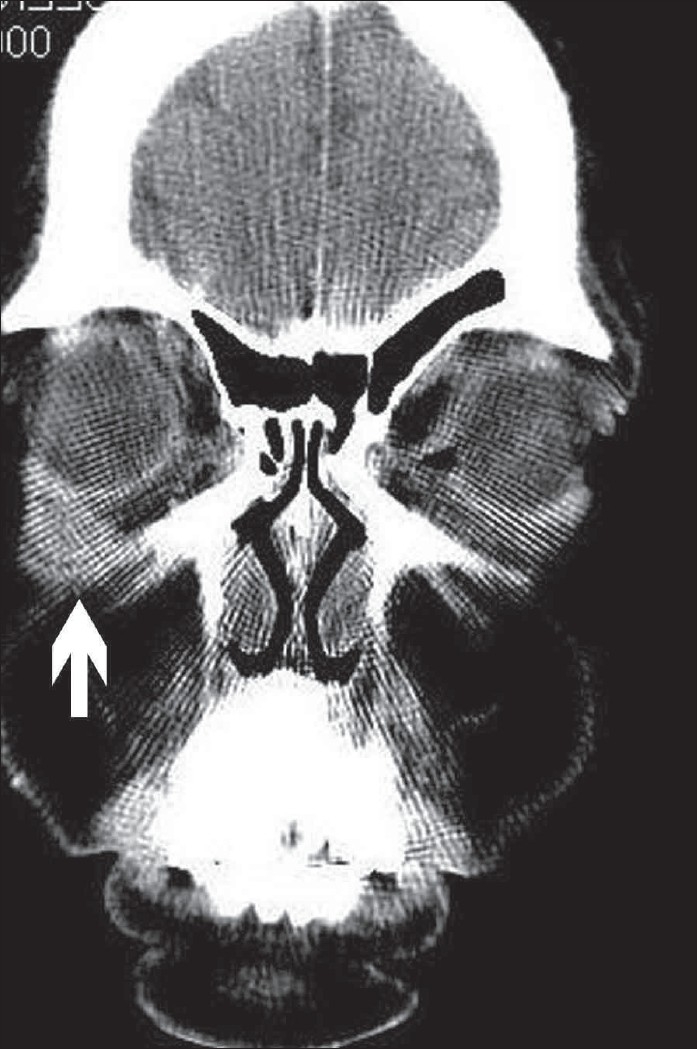
Post-contrast coronal CT image showing the same enhancing heterogeneous lesion (arrow)

A 40-year-old woman, previously asymptomatic, suddenly developed itching, redness, and edema of her right eyelid. Ophthalmological examination revealed a small, firm, painless, freely mobile swelling in relation to the right inferior orbital margin. On aspiration of the swelling, pus was obtained and we made a clinical diagnosis of an abscess. Since the lesion persisted despite antibiotic treatment, the patient was sent for a CT scan of the orbit, which showed a well-defined, peripherally enhancing, heterogeneous soft tissue lesion, measuring 1.0 × 1.0 × 1.0 cm, in the right inferior orbit, extraconal in location [Figures 1–3]. The globe, extraocular muscles, retroorbital fat, and optic nerve were normal.

The differential diagnosis entertained at this point included orbital abscess, inflammatory pseudotumor, sebaceous cyst, and metastasis from an occult primary. However, routine blood investigations and systemic examination did not reveal any abnormality.

**Figure 4:**
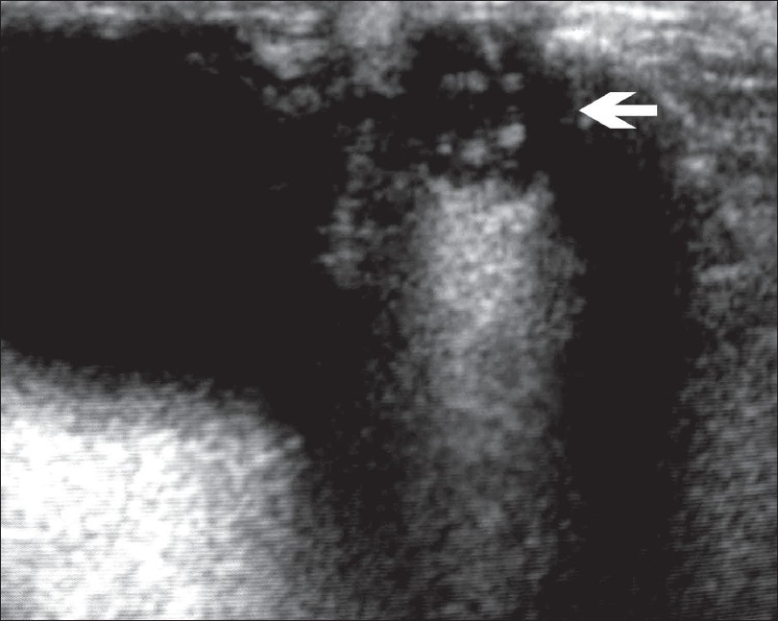
High-resolution USG image showing a parallel-walled, folded, motile, tubular structure within a cystic lesion (arrow) below the right globe

A high-resolution USG showed an actively motile, folded tubular structure with parallel echogenic walls, possibly a worm [Figure 4].

**Figure 5:**
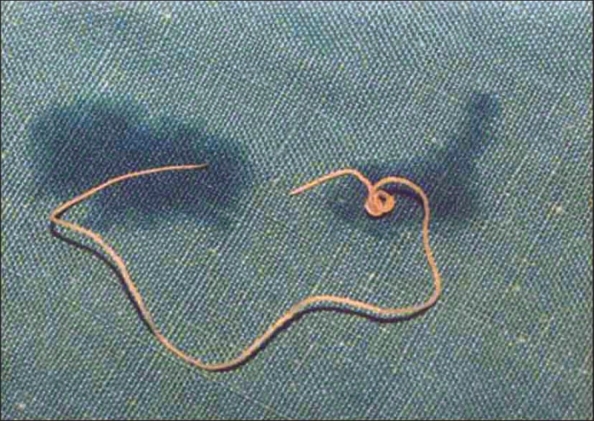
Photograph of the extracted worm

**Figure 6:**
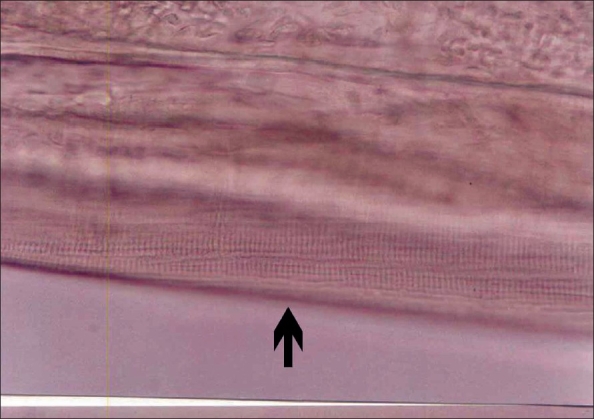
Photomicrograph of a glycerine wet mount showing the characteristic cuticular longitudinal ridges with cross-striations (beaded appearance; arrow)

Excision of the swelling done under local anesthesia, revealed a well-circumscribed, encapsulated tumor, adherent to the skin and to the orbicularis oculi. On opening the capsule, a thin thread-like, live, motile worm was noted. Microbiological examination confirmed that the parasite was *Dirofilaria repens*. The parasite was 13.5 cm long and 0.5 mm thick [Figure 5]. Microscopically, on glycerine wet mount, the characteristic cuticular longitudinal ridges with cross-striations were seen. Two reproductive tubes and a single intestinal tube were evident [Figure 6]. Histologic sections showed the characteristic multilayered cuticle and the typical longitudinal muscle cells and lateral chords. Based on the macroscopic and microscopic features and the geographic location, the worm was identified as *D. repens.* This was later confirmed by the London School of Tropical Medicine and Hygiene. The surrounding tissue showed chronic inflammatory cells, predominantly lymphocytes with a few plasma cells, eosinophils, and epithelioid cells.

## Discussion

Dirofilariasis is a worldwide helminthic zoonosis. *Dirofilaria (Nochtiella) repens* is a nematode worm belonging to Class Secernentea, Order Spirurida, and Family Onchocercidae. The number of cases of human dirofilariasis reported in the last 50 years has gradually increased and it may be described as one of the emerging zoonoses. Till date, at least 782 cases caused by *D. (N.) repens* have been reported worldwide and 372 of them have been new cases, with reports published between 1995 and 2000.[1]

The species most relevant to humans are *D. immitis, D. tenius, D. ursi, and D. repens. Dirofilaria (Nochtiella) repens* is endemic in southern-middle Europe and in some parts of Asia and Africa. Subcutaneous, ocular, and pulmonary dirofilariasis due to immature adults of either *D. repens* or *D. immitis* has been reported in humid temperate regions of both hemispheres.[2] Dirofilaria is a common parasite of dogs, who constitute the main source of infection. Humans are accidental hosts and many infected subjects are asymptomatic. Transmission occurs through the bite of zooanthropophilic types of Aedes, Culex, or Anopheles mosquitoes carrying infective larvae acquired from the microfilariae-rich blood of animal hosts parasitized with either deep-seated or subcutaneous worms of the Dirofilaria species. In humans, the nematode causes a subcutaneous or superficially located inflammatory reaction that traps it within a nodule, where it may survive for many years.[3] Occasionally, the nematode may invade the vascular system and lead to visceral, mainly pulmonary, forms of dirofilariasis. A review of all reported cases of *D. repens* dirofilariasis reveals a predilection for upper body sites (76%) over the lower body (24%). Of these, orbital lesions account for 31% of the cases.[4] Only one case of circulating diromicrofilaremia in humans has been reported in the medical literature.[5] Eosinophilia occurs in less than 15% of cases with *D. immitis* and rarely with *D. repens*.

Dirofilaria is well known to affect the eye and the adnexa. The infection may be periocular, subconjunctival, or intraocular.[6] Such lesions are always associated with moderate to severe inflammation. However it may also present as a noninflammatory lid tumor.[7] In general, the diagnosis of human dirofilariasis is based on histologic examination. Useful characteristics for differentiating between the different Dirofilaria species, are the size and the features of the body wall, i.e., thickness of the cuticle and its structure, ridges, lateral chords, and number and type of muscle cells.[8] Precise identification of Dirofilaria species may be achieved with DNA analysis based on the polymerase chain reaction.[9]

Therapy with systemic antibiotics has proved useless and surgical removal of the worm is the only known treatment. Usually, the clinical symptoms disappear after the parasite is removed and no adjunct therapy is necessary.

Although the incidence of human subcutaneous dirofilariasis has been increasing over the last 5 decades,[1] the imaging features of dirofilariasis are not well known. MRI findings have been described in a single case[10] and include the visualization of an enhancing thick-walled, semiliquid structure with a discrete, tubular, central signal, representing the worm on T1W images. However, even in that case, the diagnosis of a worm could be made only after surgical excision. Thus, dirofilariasis should also be included in the differential diagnosis of an orbital swelling with inflammation, especially in endemic areas.[6] The uniqueness of our case lies in the fact that we could detect the presence of the worm with USG. Once again, this case demonstrates the potential of high-resolution USG in providing dynamic, real-time imaging, even in this era of newer and more sophisticated imaging modalities.
